# A Different Perspective on the Characterization of a New Degradation Product of Flibanserin With HPLC–DAD–ESI-IT-TOF-MS^n^ and Its Pharmaceutical Formulation Analysis With Inter-Laboratory Comparison

**DOI:** 10.1093/jaoacint/qsad074

**Published:** 2023-06-22

**Authors:** Aysun Geven, Saniye Özcan, Serkan Levent, Nafiz Öncü Can

**Affiliations:** Anadolu University, Faculty of Pharmacy, Department of Analytical Chemistry, 26470 Eskisehir, Türkiye; Anadolu University, Faculty of Pharmacy, Department of Analytical Chemistry, 26470 Eskisehir, Türkiye; Anadolu University, Faculty of Pharmacy, Central Analysis Laboratory, 26470 Eskişehir, Türkiye; Anadolu University, Faculty of Pharmacy, Central Analysis Laboratory, 26470 Eskişehir, Türkiye; Anadolu University, Faculty of Pharmacy, Department of Pharmaceutical Chemistry, 26470 Eskisehir, Türkiye; Anadolu University, Faculty of Pharmacy, Department of Analytical Chemistry, 26470 Eskisehir, Türkiye; Anadolu University, Faculty of Pharmacy, Central Analysis Laboratory, 26470 Eskişehir, Türkiye

## Abstract

**Background:**

Flibanserin (FLB) was first synthesized as an antidepressant drug; however, due to its enhancing effects on sexual activity, it was approved for treatment of hypoactive sexual desire disorder in women in 2015.

**Objective:**

The aim of this study was to develop a new and fully validated HPLC method for analysis of FLB in pharmaceutical formulations besides its degradation products, and identification of possible formation mechanisms by using HPLC-DAD-ESI-IT-TOF-MS^n^.

**Method:**

The HPLC separation was achieved in a Supelco Ascentis^®^ Express series phenyl hexyl column (100 × 4.6 mm, ID 2.7 µm). The mobile phase was acetonitrile–ammonium acetate solution (50:50, v/v, 10 mM, pH 5.4) mixture, which was pumped at the rate of 0.5 mL/min. Chromatography, detection, and structural identification was performed by using a LCMS-IT-TOF instrument (Shimadzu, Japan).

**Results:**

1–(2-(4–(3-hydroxy-5-(trifluoromethyl)phenyl)piperazine-1-yl)ethyl)-1,3-dihydro-2H-benzo[d]imidazol-2-one is proposed as a novel degradation product, with a mass of 407.1695 and a formula of C_20_H_21_F_3_N_4_O_2_ with a margin of error about 0.001 ppm. The developed method is applicable with 98% accuracy within the 2.5–50.0 µg/mL range. The LOD and LOQ were about 500 ng/mL and 1.50 µg/mL, respectively. The transferability and variation between laboratories were tested by inter-laboratory comparison and evaluated with one-way analysis of variance.

**Conclusions:**

A novel FLB degradation product, which was produced under oxidative forced degradation conditions was observed and identified for the first time; in addition, the formation kinetics of the degradation product besides decomposition of FLB was studied. Furthermore, an inter-laboratory comparison was carried out, and application of the proposed method on a pseudo Addyi^®^ (Sprout Pharmaceuticals, Inc.) sample was tested using both instrument configurations.

**Highlights:**

A novel stability-indicating assay method was developed and fully validated according to the International Council on Harmonization (Q2) R1 for the analysis of FLB in the pharmaceutical preparations. A new degradation product was identified in the oxidative forced degradation condition and characterized using HPLC–DAD–ESI-IT-TOF-MS^3^. Moreover, the possible mechanism and the formation kinetic of the degradation product were revealed. In addition, the developed method was transferred to another LC-PDA instrument for inter-laboratory comparison. Finally, the current method was applied to a pseudo formulation of Addy in both instruments, and ANOVA was applied for evaluation.

While 43% of women have been affected by sexual problems at some point in their lives, approximately 22% of them report that they have personal problems related to sexual intercourse (1). Common sexual problems include sexual pain, arousal problems, orgasmic dysfunction, and low or missing desire. Hypoactive sexual desire disorder or sexual dysfunction in women is characterized by a lack of sexual fantasies and an inability to desire sexual activity that is not explained by medication (recreation or prescription), psychiatric (depression), or other sexual conditions, causing marked distress and/or interpersonal difficulty as defined in the American Psychiatric Association’s *Diagnostic and Statistical Manual* (2, 3). The IUPAC name of flibanserin (FLB) (CAS no. 167933-07-5) is 3-[2-[4-[3-(trifluoromethyl)phenyl]piperazin-1-yl]ethyl]-1H-benzimidazole-2-one in which the amino proton is replaced by a 3-(trifluoromethyl)phenyl moiety. Its molecular formula is C_20_H_21_F_3_N_4_O, and its molecular weight is 390.04 g/mole. Due to its chemical structure, it behaves as a weakly acidic compound with a pKa value of about 5.9. Log K_OW_ and log P values are reported as 3.4 and 4.3 (4, 5).

LC-MS/MS is the dominant technique, and C_18_ bonded silica-based particle columns are the most widely used stationary phases in the previously reported methods (6–10); all the columns used in these assays are relatively short, narrow, and small particle columns produced with recent technologies. Moreover, there is also a limited number of HPLC studies on FLB analysis in which detection was absorbance-based (*see*Supplemental Table S1); no internal standard was used in these methods (11, 12), and analyses were performed by using classical C_18_ particle columns (11,13–15). On the other hand, there are some other applications in the literature, in which different analytical techniques were used: analysis of Flibanorin^®^ film-coated tablets and human plasma using thin-layer chromatography (16), first-derivative spectrophotometric methods for determination of FLB in Veroxeserin^®^ tablets in the presence of its oxidative degradation products (17), second-derivative synchronous fluorimetric method for determination of FLB and sitagliptin phosphate in synthetic mixtures and human plasma samples (18), and characterization of *N*-oxide metabolite by LC-MS and NMR spectroscopy (19). Moreover, voltammetric determination of FLB in bulk form, Flibanorin tablets and human plasma (20), spectrofluorometric methods for FLB determination in Veroxeserin and human urine samples (21). An RP-HPTLC method for the quantitation of FLB commercial film-coated tablets (22) also was reported previously.

The novelty of the study presented herein is the identification of a novel degradation product, which was produced under oxidative forced degradation conditions, and comprehensive description of its formation mechanism with high-resolution LC-MS^n^ studies. Compared to the methods in the literature, this study describes a specific and robust analytical procedure for compendial applications, thanks to its valuable features of being accurate, precise, and rugged for routine analyses on finished products and production processes. Analytical method validation was conducted according to the Q2(R1) Guideline of the International Council for Harmonization of Technical Requirements for Pharmaceuticals for Human Use. One of the valuable features of the developed method is its stability-indicating nature. The novel degradation product of FLB was fragmented using MS^n^ technique in a HPLC–DAD–ESI-IT-TOF-MS^n^ instrument, its molecular structure was characterized, and its formation mechanism was explained in detail. The method is simple and easy to apply in pharmaceutical analytical R&D laboratories, and it also uses features of core-shell column technology.

## Experimental

### Chemicals and Reagents

Analytical grade chemicals, i.e., ammonium acetate, sodium hydroxide, hydrochloric acid, hydrogen peroxide, and acetic acid, and HPLC grade solvents, i.e., acetonitrile and methanol, were purchased from Sigma-Aldrich (USA). FLB's purity is 99.9% (w/w), and its hydrogen chloride salt was purchased from Toronto Research Company (Canada).

### Instruments

A Nexera-i series LC-2040C 3D liquid chromatograph from Shimadzu (Japan) was used for regular HPLC analyses. The software of the system was LabSolutions 5.81 (Shimadzu), which was installed on a Windows 10 based system.

The inter-laboratory comparison study was gained with Shimadzu LC-MS/MS 8040: Nexera XR Series, connected with a CBM-20A communications bus module, LC-20AD gradient pump, DGU-20A3R degasser, SIL-20AC autosampler, CTO-10ASVP column oven, and SPD-M20A PDA detector. The HPLC system had LCSolutions 1.11 SP1 software.

The LCMS-IT-TOF series liquid chromatography–high-resolution mass spectrometry instrument (Shimadzu) was used for structural characterization; the instrument consisted of the following modules: DGU-20A3 degasser, 2× LC-20AD gradient pump, SIL-20A autosampler, CTO-10ASVP column oven, CBM-20A communication module, and ion-trap and time-of-flight (IT-TOF) mass spectrometer. LCMS Solutions 3.80 software was used for setting instrumental parameters and spectrum integration.

Other auxiliary laboratory devices used in the preparations were the XSE 105 Dual Range model analytical balance and SevenMulti model pH meter from Mettler Toledo (Switzerland), RK 100 H model ultrasonic bath from Bandelin (Germany), Reax Top model vortex-mixer from Heidolph (Germany), and Rotina 380 R refrigerating centrifuge from Hettich (Germany), stability cabinets from KRC Laboratory Instruments (Türkiye), and Research model pipets from Eppendorf (Germany).

### Instrumental Parameters

In HPLC analyses, the flow rate of the mobile phase was 0.5 mL/min, and the column temperature was set as 40.0 ± 0.1 °C. The autosampler thermostat temperature was fixed at 15.0 °C to maintain the stability of the sample and reference solutions, and the injection volume was determined as 1.0 µL. In addition, the optimized method conditions in HPLC were precisely transferred to a LCMS-PDA system for inter-laboratory comparisons.

Since the maximum absorbance of the FLB was detected at 204 nm, the photodiode array detector in the HPLC system was set to this wavelength. In addition, the spectra were monitored between 190 and 380 nm at a data sampling frequency of 1.5625 Hz and a time constant of 0.640 s.

High-resolution mass spectrometric analyses were performed using a hybrid IT-TOF mass spectrometer with an ESI interface (LCMS-IT-TOF). Analysis conditions were set as follows: 1.5 L/min nebulizing gas flow, 3.5 kV high voltage probe, drying gas pressure of 200 KPa, heat block temperature of 200°C, and CDL temperature of 200°C. CID parameters are 50% for the collision gas parameter, 50% for CID energy, and argon gas for CID. Also, the detector voltage of the TOF was set to 1.6 kV. The IT-TOF system was calibrated with sodium trifluoroacetate solution.

The analytical response for both the MS detector and the PDA detector system is taken as the area of the active substance signal in the chromatograms. The inter-group closeness of the results was examined by one-way analysis of variance (ANOVA). Statistical calculations were done with the GraphPad Prism v.6.0 program.

### Preparation Solutions

All stock solutions and working solutions of reference standards were prepared in methanol due to the solubility problem of FLB. Stock solutions of FLB were prepared by first dissolving 2.5 mg in methanol to obtain a 50 µg/mL solution. Further dilutions for calibration, LOD, LOQ, and quality (QC) solutions were prepared by making necessary dilutions from the stock solutions.

The forced degradation conditions to which the active substance was exposed were designed to include thermolytic, acidic/basic, hydrolytic, and oxidative stress conditions, as presented in the International Council on Harmonization (ICH) Q2(R1) guideline; concentration of degradation solutions in these tests was 17.5 µg/mL FLB (in methanol). The prepared forced degradation conditions are 1 N NaOH, 1 N HCl, 3% H_2_O_2_ (v/v), UV light for 6 h, 75% humidity for 1 h, and 1 h in 10°C increments at a constant temperature of 60°C.

FLB recovery studies are planned for its pharmaceutical preparation Addyi^®^ of Sprout Pharmaceuticals, Inc. (which contains 100 mg of FLB per tablet). However, Addyi (the only approved pharmaceutical preparation on the market that includes FLB) could not be purchased due to its prohibition in Türkiye by official regulations. Therefore, its pharmaceutical formulation was prepared in vitro, and recovery studies were carried out using this preparation. The prepared pseudo tablet formulation of Addyi per tablet contained FLB hydrochloride (50.0 mg), lactose monohydrate (143.4 mg), microcrystalline cellulose (47.81 mg), hydroxypropyl methylcellulose (2.5 mg), carboxymethyl cellulose sodium (5.0 mg), magnesium stearate (1.25 mg), and macrogol (5.5 mg); the formulation was prepared according to information given in the literature (23). Inactive ingredients were weighed for the amounts given, and further QC solutions were prepared in methanol in 100 mL volumetric flasks. The prepared mixture was thoroughly mixed in a vortex for 10 min and then filtered through PTFE (25 mm ID, 0.22 µm pore size, Isolab, Germany) syringe filters. The resulting filtrate was diluted by 1/10, and recovery solutions were prepared by diluting this solution with methanol in the required proportions.

The chromatographic separation was performed in isocratic elution mode. The mobile phase was a mixture of acetonitrile–ammonium acetate buffer (10 mM, pH 5.4) in a ratio of 50:50 (v/v*).* The buffer solution was obtained by dissolving 770.0 mg of ammonium acetate in 1 liter of water. The prepared solution was left in an ultrasonic bath for 15 min to dissolve and then filtered through nonsterile cellulose acetate membrane filters (47 mm ID, 0.45 µm pore size, Germany) made by Sartorius.

## Results and Discussion

### Investigation of the Spectral Properties of FLB in the UV-Visible Region

**Figure 1.**
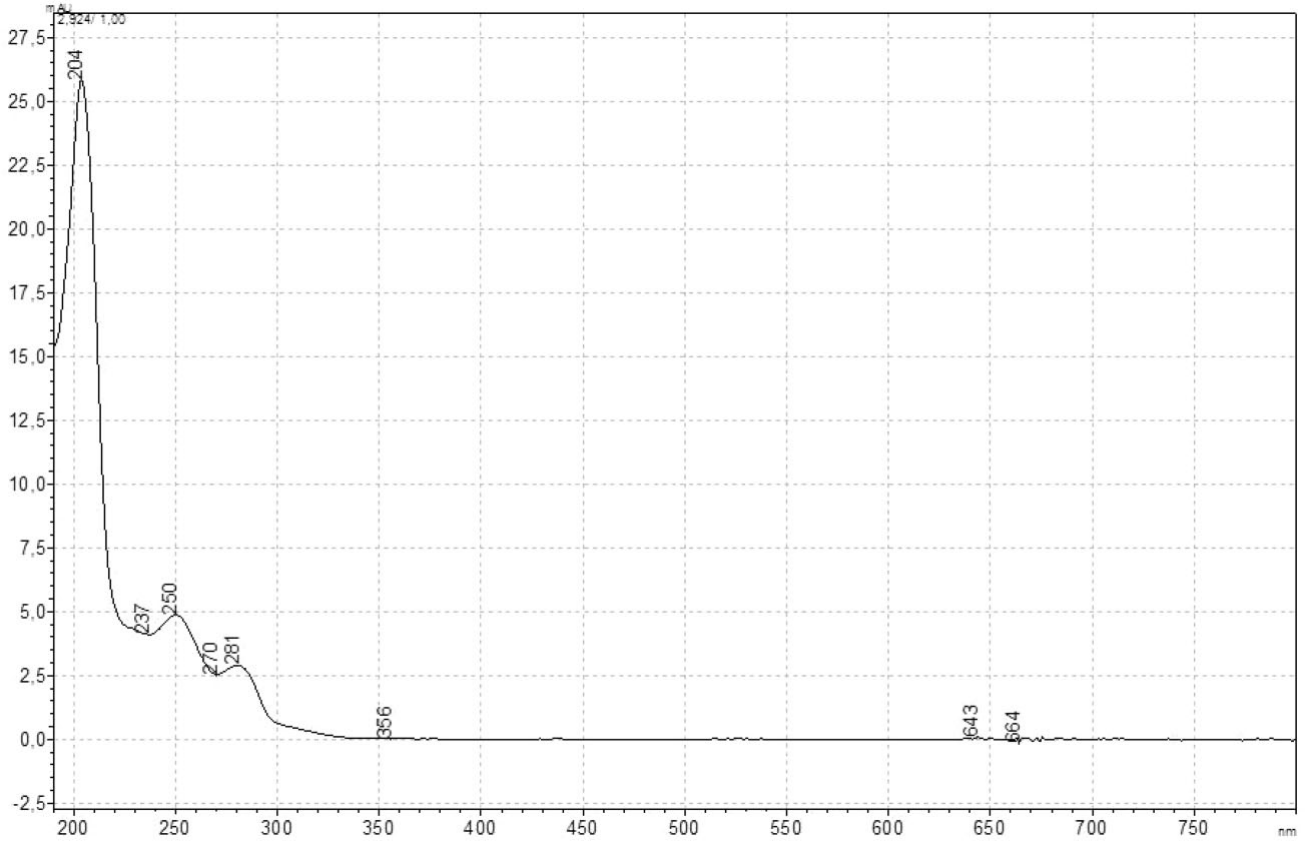
Spectrum of FLB in the UV-visible region (C = 17.5 µg/mL).

The UV spectrum of the standard FLB solution is given in Figure 1. Accordingly, the absorbance maxima of the solutions were at three points: 204, 250, and 281 nm. The maximum absorbance of the piperazine and ethylenediamine chromophore groups was observed at approximately 195–200 nm, whereas the imidazole group had maxima about 200–205 nm. Since the solvent in the experiments was methanol, it generally lowered the energy of the orbitals of the chromophore groups, causing bathochromic shifts (due to polarity). The main reason for the shift is that the solvent lowers the energy of the n orbital more than the π* orbital, and it is specially controlled by the energy of the H-bond with the n orbitals of the chromophore groups (24). In the FLB structure, with the combination of ethylenediamine and piperazine groups, a resistance against the bathochromic effect emerged, and a maximum absorbance was observed at 204 nm, and its intensity was quite high against other wavelengths. The coexistence of both chromophore groups and their location in the middle part of the FLB enabled them to absorb in a sheltered area and to gather these absorbance intensities. Thus, a very strong absorbance at 204 nm was observed, freed from the bathochromic effect of the solvent. The other chromophore group that contributes to the intensity of this absorbance is the imidazoline group, whose interactions are limited by the rigidity of the structure. The absorbance at other lower wavelengths and intensities was the hypsochromic shift of the absorbance at 274 nm, which occurs with the n* electronic transition of the carbonyl in the structure to 281 nm. Apart from this, it is caused by electronic transitions made by other chromophore groups, such as benzene, in the structure (25).

### Optimization of Chromatographic Condition

In the study, an internationally valid method was developed for the analysis of FLB using HPLC. All degradation products encountered in the analyses were attempted to be obtained by degrading the active substance under forced degradation conditions in order to make the method selective in the PDA detector. Then, chromatographic separation and analysis of FLB were performed in the presence of these degradation products. In addition, a liquid chromatography–high-resolution mass spectrometer LCMS-IT-TOF instrument was used to estimate possible formation mechanisms and qualitatively determine the obtained degradation products. To prove the reproducibility of the method, an inter-laboratory comparison study was conducted. For this purpose, the LC-PDA-MS/MS instrument, which is the second liquid chromatography device available and which has a PDA detector and at the same time analyzes with a mass detector, was used.

In the study, the chromatographic separation of FLB in the presence of its degradation product was achieved with a Supelco Ascentis^®^ Express phenyl hexyl column. It provides a high peak area and elution at an appropriate time.

The organic phase component of the mobile phase was chosen as acetonitrile due to pressure problems. For buffer selection, ammonium formate and ammonium acetate were tested at various concentrations, and pH and system suitability test (SST) parameters were calculated (23). The U.S. Food and Drug Administration (FDA) and the United States Pharmacopoeia (USP) recommend performing SST to prove the accuracy and precision of a HPLC or UPLC method. These tests are part of the method and can be done before or during validation. It can also be thought of as the features or capacity offered by a method. Tailing factor (T), resolution (R_s_), number of theoretical plates (N), capacity factor (k′), and selectivity factor (α) are the main system suitability parameters (23, 26). Accordingly, it was observed that the retention time and peak height were more affected by pH and buffer concentration, whereas the peak area remained the same, which means sharper peaks were obtained. This effect is 0.6 min at retention time and 2000 Abs at peak height for each 10 mM concentration of ammonium formate or 1 unit pH increase. However, the concentration and pH changes of ammonium acetate are 0.07 min on time and 1000 Abs at peak height. As a result, ammonium acetate was determined to be 10 mM (pH 5.1), with the most suitable SST parameters and better resistance to change. After the preliminary studies mentioned above, SST parameters, which are the first step of method validation studies, were calculated according to the USP method, and the results are given in Table 1 (23).

**Table 1.** SST data obtained for FLB analysis

	Observed value		Acceptance criteria
Parameter	HPLC	LC-PDA	
Retention time (min) ± CI^a^	5.90 ± 0.01	6.35 ± 0.02	–
Precision retention time RSD, % (n = 6)	0.13	0.34	RSD ≤%1
Injection precision for peak area RSD, % (n = 6)	0.28	0.78	RSD ≤%1
Injection precision for retention time RSD, % (n = 6)	0.01	0.01	RSD ≤%1
Tailing factor (T)	1.13	1.13	T ≤2
Capacity factor (k)	2.45	2.47	2<k < 10
Number of theoretical plates (N)	15688	15255	N > 2000
USP width	0.21	0.21	≤1
HETP (USP)	11.99	9.85	–
Resolution	24.0	21.9	1.5<

a CI = 95% confidence level.

### LC-MS-IT-TOF Method Development

Until recently, there were two methods used in the qualitative analysis of molecules: NMR and IR spectroscopy. However, advancing mass spectroscopy technology has introduced new tandem mass analyzers to the scientific community, making use of ion trap (IT) and time-of-flight (TOF) mass spectrometry. With its high-resolution mass spectrometer (HRMS), the current device provides a mass at a scale of 0.0000. In addition, the daughter ions selected with the MS^n^ technique are captured by the detector, and the smaller daughter ions are fragmented to reveal the structure of large molecules with high accuracy. This stands for revolutionary innovation in qualitative analysis. Thus, qualitative analysis is freed from the influence of traditional spectroscopic methods that require purification or samples at the mg level. Of course, the precision of traditional methods (NMR, IR) in the determination of structure is undeniable.

In the aforementioned study, HPLC analyses were performed in the presence of FLB degradation products, and one degradation product was detected under oxidative conditions. The obtained degradation product was determined and characterized by transferring the HPLC conditions to the one-to-one LCMS-IT-TOF system.

To examine the degradation behavior of FLB, solutions were prepared in various environments, and controlled heat treatment at 60°C was applied at the same time to accelerate the degradation process. Any degradation compound was not observed in the chromatogram of the active substance solution when the only temperature was applied. Moreover, it has been observed that FLB is quite stable against UV light, acidic and basic environments, and moisture and does not give any degradation products. The results obtained here are similar to the literature, because no degradation product has been found in the stability-indicating studies of FLB until today (11, 21, 22).

**Figure 2.**
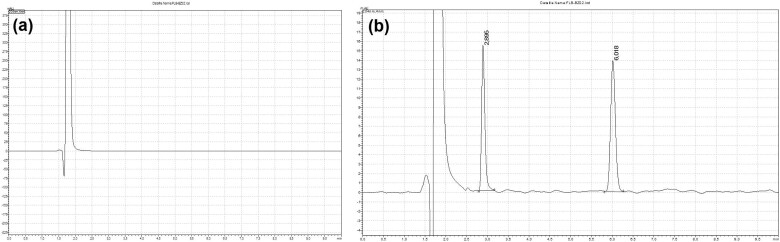
Chromatogram obtained from blank (a) and FLB solution (b) under oxidation conditions.

In the analysis of forced degradation conditions, it was observed that FLB was quite unstable under oxidation conditions. This prediction is supported by the fact that the substance does not react in temperature application or degradation under oxidation conditions in the study. Notably, the same degradation product under oxidation conditions was obtained at room temperature. However, the amount of degradation product obtained under oxidative conditions at room temperature is approximately two times less than the temperature application, and the FLB lost only about 20% in the 2 h exposure period. As shown in Figure 2a, no peak was observed in the chromatogram of the oxidation conditions’ blank solution. However, the degradation product is clearly visible in the chromatogram obtained from the exposure of the active substance in Figure 2b under forced degradation conditions in the oxidative environment by applying a temperature of 60°C.

**Figure 3.**
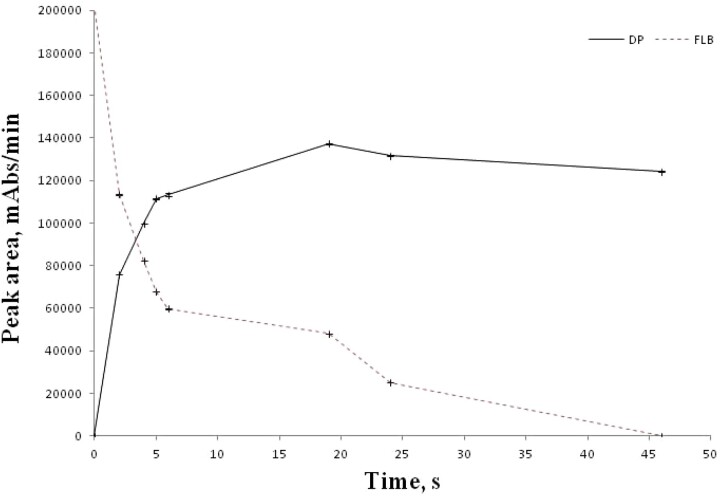
The degradation kinetic for FLB and formation kinetic for DP.

The formation kinetic of the degradation product obtained under oxidative conditions was analyzed in detail. For this purpose, samples were taken from the FLB oxidative degradation solution at intervals of 2 h and injected into the HPLC system by making a one-fifth dilution. The variation of the peak areas of the FLB and the degradation product from the obtained chromatogram according to the degradation time of the solution is given in Figure 3. According to this graph, 43.1% of FLB degraded between 0 and 2 h and 64.6% between 0 and 5 h under oxidative conditions. Afterwards, the rate of degradation of FLB started to decrease, and at the end of 46 h, only 1.8% of FLB remained in the medium.

**Figure 4.**
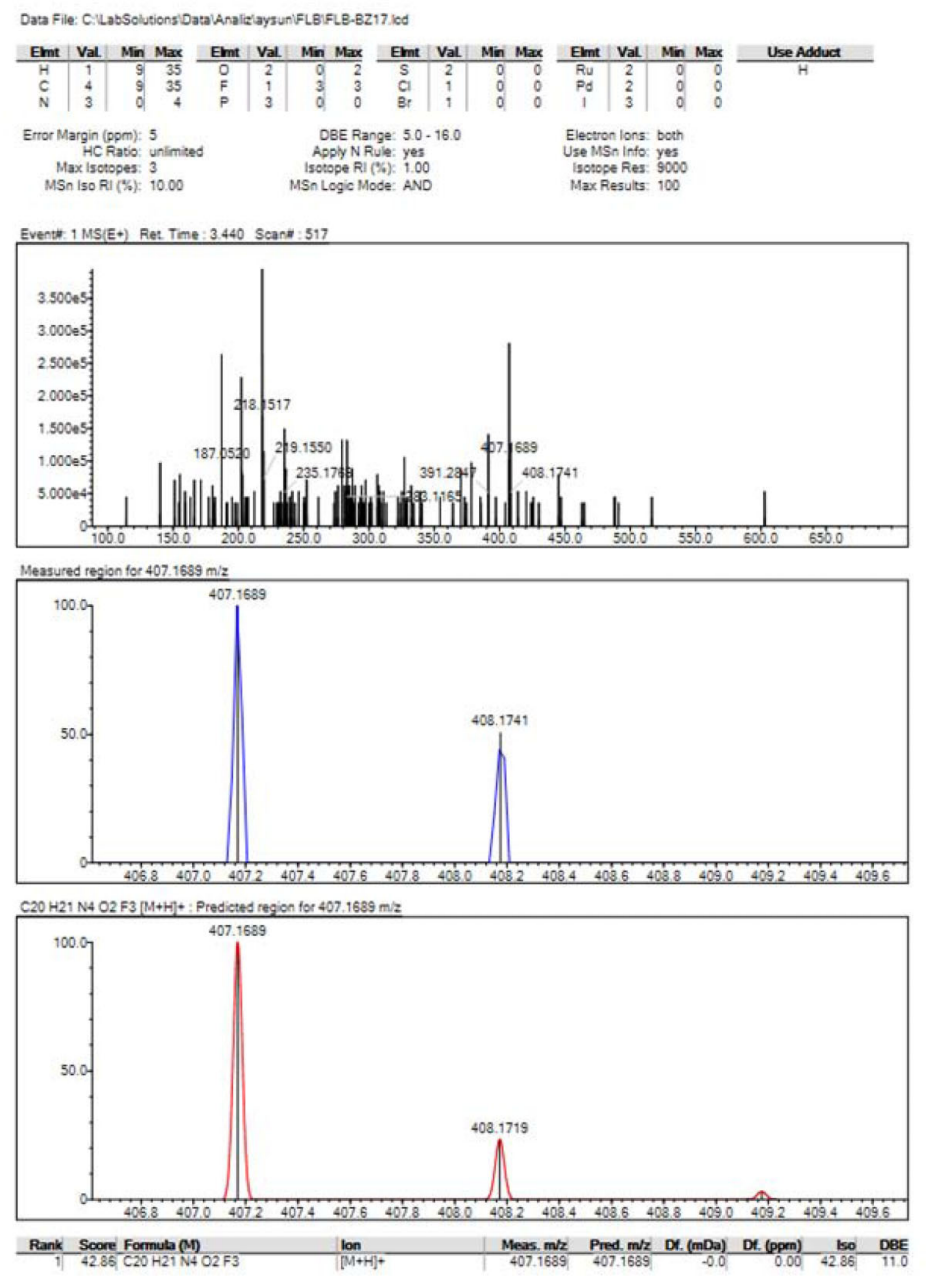
High-resolution spectra of DP.

Based on the mass spectra of the oxidative degradation conditions obtained by LCMS-IT-TOF, the new degradation product with a molecular weight of 406.41 g/mol was identified by CID fragmentation with *m/z* 407.1689 as the main ion product. The high-resolution spectra of DP are given in Figure 4.

### MS^n^ Investigations

**Figure 5.**
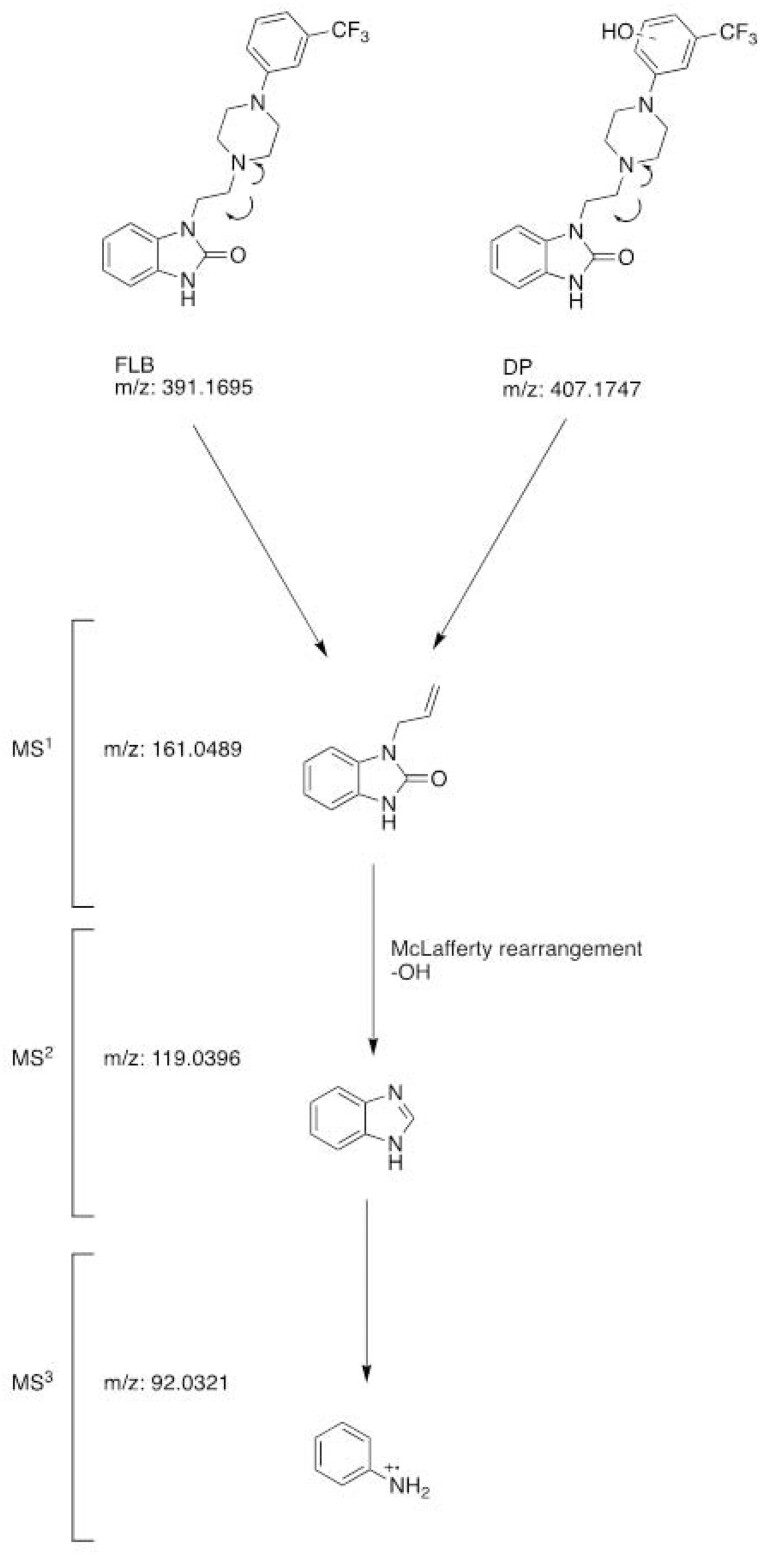
The degradation pathway and MS^n^ characterization of DP.

Correct mass ions (MS^n^) can be used to determine molecular structure. An algorithm is developed to analyze whether the elemental composition of the daughter ion can be combined to produce viable elemental compounds of the entire molecule, excluding molecular formulas that are not possible from low molecular fragments. HRMS drug screening uses fragmented software products that use general fragmentation rules to generate fragments based on the structure of a compound and identify the characteristic daughter ions in it. A study is a practical approach to preliminary standard definition in a large full-mass database without the need for reference standards. Triple-quad and hybrid linear IT MS^3^ or MS^4^ provide the highest amount of daughter ion data but suffer from many false negatives and very high total time consumption, especially with MS^3^ or MS^4^. It can be used in unit-resolution mass spectrometers to monitor and identify common neutral losses, including methylation, acetylation, and glucuronidation (27). Accordingly, the predicted formation mechanism of the degradation product characterized under oxidative forced degradation conditions and the MS^n^ characterization performed are given in Figure 5. When the degradation product is examined in LCMS-IT-TOF, an excess of oxygenic mass in the structure is remarkable. The closed formula suggests the expected hydroxyl product. In the presence of FLB, hydroxylation through the aromatic ring is expected with general knowledge of organic chemistry. An MS^n^ study was carried out to determine which of the two possible aromatic ring hydroxylations took place. The FLB standard and the degradation product were analyzed under the same conditions. Accordingly, in both studies on molecular ions and the daughter ions obtained from them, the possible degradation pathway of the substance has been suggested. It has been determined that the hydroxyl is on the trifluoromethyl benzene ring since the degradation pathways are the same for the degradation product and the active substance. In MS^1^, the bond between the piperazine nitrogen and the ethylene carbon was broken heterolytically, and a product with mass *m/z* 161 was obtained. The benzimidazole compound *m/z* 119 was obtained by McLafferty rearrangement and hydroxyl removal in MS^2^. Finally, in MS^3^, the aniline compound was obtained by opening the ring, and the predicted structure for the degradation product was supported. As a result, the IUPAC name is 1–(2-(4–(3-hydroxy-5-(trifluoromethyl)phenyl)piperazine-1-yl)ethyl)-1,3-dihydro-2H-benzo[d]imidazol-2-one, and the chemical formula is proposed as a new decomposition product with a mass of 407.1695 and a closed formula of C_20_H_21_F_3_N_4_O_2_ with a margin of error of 0.00 ppm. In the literature, the proposed degradation product was found that when the activity of FLB was detected, it was thought that it could show similar activities and that the compound was synthesized and patented. However, the compound has not yet been recorded as a degradation product of FLB, and its detection was made for the first time in this study.

In the stability-indicating studies on FLB, the forced degradation conditions were carried out in four different ways: 3% H_2_O_2_ at 100°C (17), 3% H_2_O_2_ (11) and 15% H_2_O_2_ in a water bath, and 3% H_2_O_2_ (19) and 2.4% H_2_O_2_ (13) at room temperature, previously. The degradation products obtained were broken up of FLB at high temperatures under the forced oxidative conditions. In other words, the degradation conditions used in the studies were examined at a temperature of around 100°C or at room conditions. In this study, FLB was treated with 60°C and 3% H_2_O_2_. These forced conditions and the degradation product were also not available in the literature (11, 12, 14). This degradation product may have been obtained because lower temperature was applied, because, as stated by previous studies and observed in this study, the behavior of FLB in oxidative conditions is dependent on temperature. Although it is stable under oxidative degradation conditions at room temperature, it has been observed that it begins to decompose when temperature is applied. The degradation products obtained were broken up of FLB at high temperatures under the forced oxidative conditions.

### Validation Results

The calculated linearity and precision data of the proposed method are given in Table 2. In the method, possible interference effects that may affect the signal of the analyte have been eliminated by performing chromatographic separation under forced degradation conditions and optimizing the method. The method has very low LOD and LOQ concentrations, a wide working range, and a linearity coefficient of 0.999. In addition, when the results of intraday and interday linearity are examined, there is no significant difference between the data obtained. *P *> 0.05 was calculated in the analysis of variance study with one-way ANOVA. In addition, the purity of the peak in the detector was calculated at 0.999887, and it was again proven that the current peaks belong only to FLB. Thus, the specificity and selectivity of the method were examined. According to the literature, the performance of this method is among the best.

**Table 2.** Linearity and precision data of FLB

Parameter	HPLC
Linearity range	2.50–50.0 (µg/mL)
Slope ± SD^a^ (intraday, *n* = 8)	15279.9 ± 84.3
Intercept ± SD^a^ (intraday, *n* = 8)	−4955.2 ± 2315.8
Regression coefficient (intraday, *n* = 8)	0.99982
Limit of detection	500 ng/mL
Limit of quantitation	1.50 µg/mL
Slope ± SE^b^ (interday, *n* = 8 × 3 days)	15 219.0 ± 65.04
Intercept ± SE^b^ (interday, *n* = 8 × 3 days)	−5510.0 ± 1786.0
Regression coefficient (interday, *n* = 8 × 3 days)	0.99960
ANOVA	*F*(2,15) = 1.437 *P* = 0.268507 (*P* > 0.05)
Repeatability (intraday, mean, *n* = 8)	378 365.0
Repeatability (intraday, SD^a^, *n* = 8)	370.4
Repeatability (intraday, RSD^c^%, *n* = 8)	1.01
Repeatability (intraday, SEM^d^, *n* = 8)	151.2
Repeatability (intraday, CI^e^, *n* = 8)	296.4
Repeatability (interday, mean, *n* = 24)	378 578.9
Repeatability (interday, SD^a^, *n* = 24)	765.7
Repeatability (interday, RSD^c^, %, *n* = 24)	2.1
Repeatability (interday, SEM^d^, *n* = 24)	180.45
Repeatability (interday, CI^e^, *n* = 24)	353.7
ANOVA	*F* (2,21) = 0.0004 *P* = 0.999598 (*P* > 0.05)

a SD = Standard deviation.

b SE = Standard error.

c RSD = Relative standard deviation.

d SEM = Standard error of the mean.

e CI = 95% confidence interval.

After calculating the linearity and precision data of the method optimized for FLB analysis, accuracy studies were performed. For the recovery experiments, FLB was added to the hand-prepared Addyi tablet formulation solution by the standard addition method. The study was performed with nine independent assays at three different concentrations. The data obtained from the recovery experiments are presented in Supplemental Table S2. When the results are examined carefully, it is seen that the effects of processes such as dilution and filtration on the method’s accuracy are within acceptable limits. In the recovery studies, the maximum error is 3.9%, and the maximum RSD for the concentration found is 2.2%. These values are excellent for analysis at the µg/mL level.

The developed HPLC method was confirmed by inter-laboratory comparison with the Shimadzu brand LC-PDA device, with intermediate precision studies. For this purpose, calibration solutions were analyzed in the LC-PDA system under the same method conditions, and whether there was a significant difference between them was examined by one-way analysis of variance. The results obtained are shown in Table 3. In addition, the recovery solutions prepared for the accuracy studies of the HPLC method were similarly analyzed in the LC-PDA system. The FLB concentration from the peak areas in the LC-PDA system was calculated from the linear regression equation of the HPLC method. The analysis of the variance of the concentration results of the two devices for each concentration level was calculated with a single factor, ANOVA. The results obtained are given as the repeatability variance (S_r_) instead of the intermediate variance (S_I_) value since the study was conducted by the method developer. The S_r_ averages calculated from the analysis results obtained from both devices in triplicate for three concentration levels were found to be 0.2486. It can be said that the results are compatible and that the inter-laboratory comparison study was successful.

**Table 3.** Intermediate precision of FLB

Parameter	LC-PDA
Linearity range	2.50–50.0
Slope ± SE^a^ (intraday, *n* = 6)	16 091.01 ± 166.38
Intercept ± SE^a^ (intraday, *n* = 6)	−9247.23 ± 4568.87
Regression coefficient (intraday, *n* = 6)	0.99936
ANOVA	*F* (1,14) = 0.0128 *P *= 0.911194 (*P* > 0.05)

a SE = Standard error.

Robustness studies were conducted to examine in detail the variables to which the method is sensitive. The robustness data obtained in this study are in Supplemental Table S3. The investigated SST parameters of the method are given as retention time, the number of the theoretical plate, and the tailing factor. The maximum difference in tailing factor against changes in method parameters is 4.75%, which is acceptable. The number of theoretical plates, on the other hand, is very sensitive to the changed parameters. The greatest amount of change was obtained with a flow rate change of around 24%. The last change was observed when the amount of organic solvent in the mobile phase decreased. Retention time, on the other hand, did not show a significant change, only in the wavelength of the changed parameters. There are differences of up to 36% in the changes in property flow rate and organic component percentage of the mobile phase. Method sensitivity is important in liquid chromatography studies, especially when columns with smaller particles are used. Since the column used in this study has a relatively small particle size and internal volume, there are sensitivities in the method.

The stability of the standard solution of FLB prepared in methanol was analyzed after storage under different conditions. It was stored at room temperature for 24 and 48 h for short-term stability, at 20°C for three weeks to evaluate long-term stability, and in three freeze–thaw cycles, and then analyses of each solution were made. These analyses were repeated three times, and the results were evaluated as 95% confidence level recoveries and RSD results. Supplemental Table S4 shows that FLB solutions are stable under these conditions.

### Application of the Developed Method to the Samples

For analysts, every step of the method creation process is very important and ensures that there are fewer question marks in the next step. Method optimization, in fact, provides the basis for easier method validity and enables the discovery of the weak points of the method. Ensuring the full validity of the method means that it is reproducible and easier to apply. However, after all this process, the method developer’s sample application studies within the possibilities at his disposal mean that the method offered for use proves itself once again. In this study, by adopting this understanding, the commercial pharmaceutical tablet formulation of FLB, Addyi pseudo-formulation, was prepared and analyzed not only for recovery studies but also for sample applications. The applicability of both fully validated methods developed with these analyses has been demonstrated. The analysis results obtained are given in Supplemental Table S5.

## Conclusions

A novel stability-indicating HPLC method for the quantitative analysis of FLB in the presence of its degradation products was developed in the study. All analytical and instrumental parameters of the method have been optimized and fully validated according to the ICH regulations. The method is especially suitable for pharmaceutical formulation analysis, and a candidate for compendial methods. Moreover, a new degradation product, which occurs under oxidative conditions and had not been discovered to date, was discovered. The degradation product was characterized by the MS^3^ using LCMS-IT-TOF, and its possible formation mechanism and structure were also revealed. Furthermore, the developed method was transferred to another LC-PDA instrument, an inter-laboratory comparison study was made, and a pseudo formulation of Addyi was analyzed on both LC-PDA and HPLC instruments.

## Supplementary Material

qsad074_Supplementary_Data

## References

[1] Dhanuka I. , Simon J.A. (2015) Expert Opin. Pharmacother. 16, 2523–2529. doi:10.1517/14656566.2015.1090426 26395164

[2] Brotto L.A. (2010) Arch. Sex. Behav. 39, 221–239. doi:10.1007/s10508-009-9543-1 19777334

[3] American Psychiatric Association (2013) Diagnostic and Statistical Manual of Mental Disorders (DSM-5^®^). American Psychiatric Pub, Arlington.

[4] Sieger P. , Cui Y., Scheuerer S. (2017) Eur. J. Pharm. Sci. 105, 82–90. doi:10.1016/j.ejps.2017.04.016 28478135

[5] National Centre of Biotechnology Information (2020) Flibanserin, CID=6918248. https://pubchem.ncbi.nlm.nih.gov/compound/Flibanserin#section=Drug-Warnings (accessed 10 July 2020)

[6] Sultan M.A. , El-Eryan R.T., Attia A.K., Eissa M.J. (2019) Biomed. Chromatogr. 33, e4545. doi:10.1002/bmc.4545 30937940

[7] He L. , You W., Wang S., Jiang T., Chen C. (2019) BMC Chem. 13, 111. doi:10.1186/s13065-019-0620-9 31463480 PMC6710871

[8] Iqbal M. , Ezzeldin E., Rezk N.L., Bajrai A.A., Al-Rashood K.A. (2018) Bioanalysis 10, 1087–1097. doi:10.4155/bio-2018-0065 29692180

[9] Trocóniz I.F. , Boland K., Staab A. (2012) Pharm. Res. 29, 1518–1529. doi:10.1007/s11095-011-0648-6 22219166

[10] Sharma M.K. , Rathod R., Sengupta P. (2020) J. Anal. Toxicol. 44, 559–569. doi:10.1093/jat/bkaa009 32020175

[11] El-Behairy M.F. , Ahmed R.M., Fayed M.A.A., Mowafy S., Abdallah I.A. (2021) New J. Chem. 45, 2620–2630. doi:10.1039/d0nj05548d

[12] Ahmed R.M. , Abdallah I.A. (2020) Spectrochim. Acta. A Mol. Biomol. Spectrosc. 225, 117491. doi:10.1016/j.saa.2019.117491 31476647

[13] Chew Y.L. , Lee H.K., Khor M.A., Liew K.B., Lokesh B.V.S., Akowuah G.A. (2022) IJPER 56, 32–42. doi:10.5530/ijper.56.1.5

[14] Sharma M.K. , Pandey K., Shah R.P., Kumar D., Sengupta P. (2021) Microchem. J. 167, 106281. doi:10.1016/j.microc.2021.106281

[15] Pulusu V. , Kommarajula P. (2022) JCCL 11, 237–244. doi:10.5267/j.ccl.2021.11.004

[16] Hosny N.M. , Abdelkarim M., Gadallah M.I., Mousa H.S. (2022) J. Chromatogr. B Analyt. Technol. Biomed. Life Sci. 1195, 123204. doi:10.1016/j.jchromb.2022.123204 35248898

[17] Ahmed R.M. , Abdallah I.A. (2020) J. Appl. Spectrosc. 87, 976–985. doi:10.1007/s10812-020-01097-w

[18] Kamal M.F. , Morshedy S., Saad D.A., Moneeb M.S. (2021) J. Chromatogr. B Analyt. Technol. Biomed. Life Sci. 1184, 122955. doi:10.1016/j.jchromb.2021.122955 34653844

[19] Sharma M.K. , Shah R.P., Sengupta P. (2020) J. Chromatogr. B Analyt. Technol. Biomed. Life Sci. 1139, 121993. doi:10.1016/j.jchromb.2020.121993 32004940

[20] Hosny N.M. , Ali M.F.B. (2022) Talanta 245, 123420. doi:10.1016/j.talanta.2022.123420 35413628

[21] Ahmed R. , Abdallah I. (2020) Molecules 25, 4932. doi:10.3390/molecules25214932 33113816 PMC7663165

[22] Foudah A.I. , Shakeel F., Alqarni M.H., Alam P. (2021) Microchem. J. 164, 105960. doi:10.1016/j.microc.2021.105960

[23] United States Pharmacopeia (USP) (2020) 43 National Formulary (NF) 38 General Chapters: <621> Chromatography, Pharmacopeial Convention, Rockville, MD, USA

[24] Hochstrasser R.M. (1968) Acc. Chem. Res. 1, 266–274. doi:10.1021/ar50009a002

[25] Kalsi P. (2007) Spectroscopy of Organic Compounds, New Age International

[26] U.S. Food and Drug Administration, https://www.fda.gov/files/drugs/published/Process-Validation–General-Principles-and-Practices.pdf (accessed April 5, 2023)

[27] Pasin D. , Cawley A., Bidny S., Fu S. (2017) Anal. Bioanal. Chem. 409, 5821–5836 doi:10.1007/s00216-017-0441-4 28634759

