# Supplementary material for: A Different Perspective on the Characterization of a New Degradation Product of Flibanserin With HPLC–DAD–ESI-IT-TOF-MSn and Its Pharmaceutical Formulation Analysis With Inter-Laboratory Comparison
Source: J AOAC Int. 2023 Jun 22;106(5):1145–53. doi: 10.1093/jaoacint/qsad074 (PMC10560319; doi:10.1093/jaoacint/qsad074)
Supplement: qsad074_Supplementary_Data [file qsad074_supplementary_data.docx]

**Supplementary Material**

**A Different Perspective on the Characterization of a New Degradation Product of Flibanserin with HPLC–DAD–ESI-IT-TOF-MS^n^ and its Pharmaceutical Formulation Analysis with inter-laboratory comparison**

**Author’s Name:** Aysun Geven^1^, Saniye Özcan^1,2^, Serkan Levent^2,3^, Nafiz Öncü Can^1,2*^

**Authors Affiliations:**

^1^Department of Analytical Chemistry, Faculty of Pharmacy, Anadolu University, 26470, Eskisehir, Turkey

^2^Central Analysis Laboratory, Faculty of Pharmacy, Anadolu University, 26470 Eskişehir, Turkey.

^3^Department of Pharmaceutical Chemistry, Faculty of Pharmacy, Anadolu University, 26470, Eskisehir, Turkey

**Orcid Numbers:**

Aysun Geven: https://orcid.org/0000-0001-5436-7519

Saniye Özcan: https://orcid.org/ 0000-0002-5492-0457

Serkan Levent: https://orcid.org/ 0000-0003-3692-163X

Nafiz Öncü Can: https://orcid.org/0000-0003-0280-518X

**Corresponding author’s e-mail:**

e-mail: [nafizoc@anadolu.edu.tr](mailto:nafizoc@anadolu.edu.tr)

**Table S1.** HPLC studies on FLB in literature

| **Column** | **Detection (nm)** | **Linearity range (μg/mL)** | **RT (min)** | **LOD (μg/mL)** | **LOQ (μg/mL)** | **App.** | **Ref** |
| --- | --- | --- | --- | --- | --- | --- | --- |
| Luna C-18 (250 × 4.6 mm, 5 µm) | 250 | 1.0-20.0 | 5.1 | 0.0109 | 0.0330 | - | (23) |
| Eclipse XDB C_18_ (150 × 4.6 mm, 5 μm) | 255 | 0.1–10.0 | 2.4 | 0.05 | 0.1 | pharmaceutical dosage | (24) |
| Eclipse C18 (250 × 4.6 mm, 5 μm) | - | 10.0-70.0 | 16.1 | 0.4 | 1.3 | - | (14) |
| Agilent C18 (150 × 4.6 mm, 5µm) | 248 | 20.0-200.0 | 2.8 | 2.45 | 7.42 | - | (15) |
| Agilent SB-C18 (100 × 4.6 mm, 1.8 µm) | 237 | 0.1–3.0 |  | 40.0 | 120.0 | Veroxeserin^®^ | (25) |

**Table S2.** Recovery values for FLB *(n=3)*

|  |  |  | **Precision** | | **Accuracy** | |
| --- | --- | --- | --- | --- | --- | --- |
|  | **Added (μg/mL)** | **Found (μg/mL)±CI^a^** | **SD** | **RSD (%)** | **Rcovery (%)** | **Bais (%)** |
| **HPLC** | 20.00 | 19. 15±0.12 | 0.11 | 0.57 | 96.10 | -3.90 |
|  | 25.00 | 25.10±0.42 | 0.37 | 1.47 | 98.91 | -1.09 |
|  | 30.00 | 29.86±0.15 | 0.13 | 0.44 | 99.64 | -0.36 |
| **LC-PDA** | 20.00 | 19.24±0.48 | 0.42 | 2.16 | 97.71 | -2.29 |
|  | 25.00 | 24.69±0.15 | 0.13 | 0.54 | 98.61 | -1.39 |
|  | 30.00 | 29.20±0.21 | 0.19 | 0.65 | 98.02 | -1.98 |

^a^ 95% Confidence level

**Table S3.** The obtained data from robustness studies for FLB *(n=3)*

| **Changed parameter** | | **Retention time (min)** | **%Difference**  **(Mean±CI^a^)** | ***N*** | **%Difference**  **(Mean±CI^a^)** | ***T*** | **%Difference**  **(Mean±CI^a^)** | **Robustness** | **%Difference**  **(Mean±CI^a^)** |
| --- | --- | --- | --- | --- | --- | --- | --- | --- | --- |
| Flow rate (mL/min) | 0.55 | 5.45 | -10.08±0.14 | 12323 | -24.03±1.09 | 1.14 | 0.59±0.34 | 22.20 | -0.02±0.01 |
|  | 0.45 | 6.65 | 9.84±0.12 | 12979 | -18.90±0.70 | 1.15 | 1.37±0.94 | 22.37 | -0.02±0.01 |
| Percentage of the organic phase | 55.0 | 4.50 | -28.92±0.18 | 10288 | -41.58±0.78 | 1.19 | 4.75±0.91 | 17.35 | -0.08±0.01 |
|  | 45.0 | 8.70 | 36.33±0.08 | 15976 | 1.81±0.44 | 1.12 | -1.07±0.70 | 27.81 | 0.05±0.01 |
| Column temperature (°C) | 38.0 | 5.90 | -2.19±0.06 | 13508 | -14.94±0.29 | 1.15 | 0.12±0.04 | 22.69 | -0.02±0.01 |
|  | 32.0 | 6.06 | 0.55±0.09 | 12729 | -20.83±0.14 | 1.16 | 1.29±0.41 | 21.61 | -0.03±0.01 |
| Detector wavelength (nm) | 206 | 5.99 | -0.004±0.02 | 13564 | -14.52±0.15 | 1.14 | 1.17±0.64 | 24.65 | 0.01±0.01 |
|  | 202 | 5.99 | -0.53±0.12 | 13551 | -14.62±0.27 | 1.14 | 1.20±0.90 | 24.17 | 0.01±0.01 |

^a^%95 Confidence interval

**Table S4.** Stability data of FLB *(n=6)*

| **Added concentration (μg/mL)** | **Short-term stability**  **(24 h. room temperature)** | | **Short-term stability**  **(48 h. room temperature)** | | **Long-term stability**  **(3 weeks, -20°C)** | | **Freeze-thaw stability (3 cycles)** | |
| --- | --- | --- | --- | --- | --- | --- | --- | --- |
|  | **Found (Mean±CI^a^)** | **Recovery (%)** | **Found (Mean±CI^a^)** | **Recovery (%)** | **Found (Mean±CI^a^)** | **Recovery (%)** | **Found (Mean±CI^a^)** | **Recovery (%)** |
| 25.00 | 25.39±0.26 | 100.5 | 25.15±0.21 | 101.2 | 25.50±0.37 | 100.9 | 25.29±0.07 | 101.2 |

^a^%95 Confidence interval

**Table S5.** Results of pseudo formulation of Addyi^®^ *(n=6)*

| **Parameter** | **HPLC** | **LC-PDA** |
| --- | --- | --- |
| Mean (mg) | 99.98 | 99.53 |
| Minumum (mg) | 98.99 | 98.50 |
| Maximum (mg) | 100.1 | 100.2 |
| Standard deviation (mg) | 0.202 | 0.70 |
| %RSD | 0.202 | 0.71 |
| SEA (mg) | 0.308 | 0.288 |
| %Bias | -0.020 | -0.47 |
| Confidence interval (95% CI^a^) | ±0.161 | ±0.565 |

^a^ 95% confidence level

**

**Figure S1.** Blank solution of Addyi^®^ pseudo formulation

******

**Figure S2.** The spiked sample of Addy^®^ pseudo formulation (*%100*)
